# Overcoming recruitment barriers through meaningful community engagement: lessons learned from a maternal-child food insecurity assessment in underserved communities

**DOI:** 10.3389/fpubh.2025.1636578

**Published:** 2025-09-01

**Authors:** Gabriela Buccini, Cristina Hernandez, Kristen N. Herlosky, Shannon Rabb, Lizette Guillen, Dodds Simangan, Jyoti Desai, Alison Brown, Juanita Chinn, Timothy Grigsby, Jason Flatt, Ana A. Baumann

**Affiliations:** ^1^School of Public Health, University of Nevada, Las Vegas, NV, United States; ^2^Nevada Partners Inc., Las Vegas, NV, United States; ^3^Kirk Kerkorian School of Medicine, University of Nevada, Las Vegas, NV, United States; ^4^National Institutes of Health, Washington, DC, United States; ^5^Washington University in Saint Louis, St. Louis, MO, United States

**Keywords:** engagement strategies, underserved communities, food insecurity, maternal-child, community based participatory research

## Abstract

**Background:**

Recruiting participants from underserved communities for research can be challenging due to multilevel barriers. We conducted a maternal-child food insecurity needs assessment within underserved communities in urban Las Vegas, Nevada, and numerous barriers were faced to recruiting participants. This study aimed to examine barriers to participant recruitment, identify practical strategies for engaging participants, and analyze these strategies guided by the Meaningful Community Engagement Framework.

**Methods:**

This case study used an ethnography approach to analyze data collection barriers and community engagement strategies employed from March 2022 to February 2023 during a maternal-child food insecurity needs assessment. Inductive and deductive qualitative coding was used to classify barriers across three socio-ecological levels (families, service, and community). Engagement strategies were mapped onto the five principles of the Meaningful Community Engagement Framework guided by social justice, equity, and trust building.

**Results:**

Eleven barriers to participant recruitment were identified. At the community level, a history of being over-surveyed created apathy toward the maternal-child food insecurity needs assessment. At the service level, overburdened clinical staff were unwilling to participate in our survey. At the family level, participants questioned the legitimacy of advertising materials, length of the survey, low incentive amount, and were fearful of connection with state or federal programs and mandated reporting. To address the recruitment barriers, fifteen practical engagement strategies were mapped out across principles of the Meaningful Community Engagement Framework: “Building trust and long-term relationships” (*n* = 4), “Listening with a blank slate” (*n* = 3), “Planning to compensate for contributions” (*n* = 3), “Community service with no strings attached” (*n* = 3), “Focus on capacity building” (*n* = 2).

**Conclusion:**

Barriers to participant recruitment emerged across families, service, and community levels, highlighting the systemic challenges to research participation within underserved populations. The successful use of practical engagement strategies leveraged the connection with trusted community organizations and individuals, securing the successful completion of the maternal-child food insecurity needs assessment.

## 1 Introduction

Community assessments are the foundation for identifying health needs, developing, and implementing targeted and effective public health programs (1, 2). For a community assessment to accurately reflect a population’s health concerns, the sample of participants used must represent the larger population (2). Achieving representative participation in research depends on the researchers’ ability to effectively engage community members to participate in the study (3). This proposition is particularly challenging when assessing vulnerable populations weary of academic research, wary of strangers, and reticent to participate in research because of demographic and sociopolitical factors (3–8). This study examined barriers to participant recruitment and discusses practical engagement strategies developed and implemented to overcome these barriers during a maternal-child food insecurity needs assessment conducted in underserved communities in Las Vegas, Nevada, in the United States (U. S.).

### 1.1 The problem: food insecurity in households with maternal-child dyads

Food insecurity is the lack of consistent access to enough safe and nutritious food for an active and healthy life (9). Maternal-child food insecurity increases the risk of adverse outcomes in pregnancy (10, 11), birth (10, 12), and infant health (13, 14). Concerns about the accessibility and affordability of quality food in the U. S. were exacerbated during the COVID-19 pandemic, increasing food insecurity among maternal-child dyads (10). In 2023, 8.9 percent of U. S. households with children were food insecure (15). The percentage of food-insecure children was higher than the national average among Black or Hispanic households (14.0 percent) (15).

Mitigating maternal-child food insecurity is complex because of the association with multiple structural factors and hardships, such as poor access to healthcare, lack of transportation and childcare, and unemployment (10). Therefore, addressing maternal-child food insecurity would require interventions at multiple levels, such as community (e.g., policy changes, social protection programs), service (e.g., access to healthcare, childcare), and individual (e.g., nutrition-focused counseling) (16). Packaging and optimizing the implementation of these interventions to tackle the drivers of maternal-child food insecurity in underserved communities requires transformative research approaches (17).

Transformative research approaches require cross-sector collaboration between researchers, community members, community organizations, clinicians, health practitioners, public health agencies, and policymakers (18, 19). Bottom-up approaches to create meaningful cross-sector collaboration ensure that culturally and contextually appropriate research is conducted to achieve systemic change around the structural determinants of health (20). These research findings should be translated into sustainable community- and system-level changes (21). However, examples of how to meaningfully engage with underserved communities to promote equity are scarce (4–6), especially in the area of addressing maternal-child food insecurity.

### 1.2 The intervention: the early responsive nurturing care for food security (EARN-FS project)

The Early Responsive Nurturing Care for Food Security project (EARN-FS) was created in 2021 by a research team from a minority-serving university (University of Nevada Las Vegas, UNLV) in response to a call from the Office of the Director of the National Institutes of Health to support transformative interventions that address health disparities and advance health equity (22). The EARN-FS partnered with a well-established organization in Clark County, Nevada (i.e., Nevada Partners Inc.), which led a community-driven social intervention named the “West Las Vegas Promise Neighborhood” (WLVPN). At the time, the WLVPN intervention coordinated over 50 multi-sector partners in health, education, employment, housing, and social justice to address social determinants of health. The WLVPN targeted five zip code areas (89,101, 89,106, 89,030, 89,031, 89,032) that encompass 256,114 inhabitants, predominantly Hispanic/Latino (50%) and African American (23.6%) (23). These underserved areas corresponded to historically redlined communities (24) and had the highest risk of food insecurity in the county (25, 26). However, the WLVPN lacked focus on maternal-child health, making our community-academic partnership well-placed to co-create multilevel interventions to promote equitable practices for maternal-child food insecurity among these underserved communities.

### 1.3 The principles underlying the EARN-FS project: community-based participatory research and meaningful community engagement framework

The EARN-FS project used a community-based participatory research (CBPR) approach as an underlying principle. CBPR is an approach in which the community and researchers contribute their research expertise to improve health outcomes and quality of life and to effect positive community change (27). Turin et al. (28) described the Meaningful Community Engagement Framework, which outlines six principles to engage underserved communities: (a) no ‘parachute in and parachute out’, (b) not missing the mass population, (c) listening with a blank slate, (d) community service with no strings attached, (e) focus on capacity building, and (f) planning to compensate for contributions (28). These principles are critical to enhancing implementation efforts, including adoption and scale-up (29). Thus, combining CBPR with the Meaningful Community Engagement Framework is an approach to enhance action-based pragmatic research and close the evidence-to-practice gap (29).

Following a key recommendation for implementation success, the EARN-FS project conducted a pre-implementation community needs assessment with the goal of documenting existing assets, disparities, and gaps in maternal-child health in the WLVPN (2). During 12 months of data collection, the EARN-FS research team documented several challenges in recruiting participants, such as individuals not attending data collection meetings or not responding to survey recruitment advertisements. When facing barriers to engaging the community, the EARN-FS research team searched for literature to identify possible solutions and found very few case studies (3–8), with none specific to maternal-child health research. Documenting and reflecting on the challenges maternal-child health researchers may face in recruiting and implementing evidence-based interventions in underserved communities was crucial due to the increased federal funding for maternal health and nutrition security in recent years (22). This case study aimed to (a) examine barriers to recruiting participants, (b) identify practical strategies used for engaging participants during a maternal-child food insecurity needs assessment within underserved communities, and (c) analyze these strategies using the principles of the Meaningful Community Engagement Framework.

## 2 Methods

### 2.1 Study design

This case study used an adapted ethnography approach (30) to analyze barriers to participant recruitment and engagement strategies used from March 2022 to February 2023 during a maternal-child food insecurity needs assessment. We followed the Consolidated Criteria for Reporting Qualitative Studies (COREQ) to report this study (31).

### 2.2 Study setting

This study took place in the context of data collection for a maternal-child food insecurity needs assessment. The needs assessment research protocol consisted of a mixed-methods data collection to gather both quantitative and qualitative data and was approved by the University of Nevada, Las Vegas (UNLV) Institutional Review Board (IRB) (#1801320).

Data collection methods consisted of in-person and virtual surveys, interviews, and asset mapping. Table 1 outlines the maternal-child food insecurity needs assessment data collection strategies, including its goal, methodological approach, participation incentive, target population, sample size, outcomes, timeline, and analytical approach. Traditional outreach strategies, such as street outreach, community event attendance, flyer distribution, and paid social media advertisement, were used to disseminate the maternal-child food insecurity needs assessment.

**Table 1:** Community needs assessment data collection: goal, approach, target population, sample, and completed responses.

Data collection methods	Goal	Methodological approach	Incentive (yes/no)	Target population	Sample	Projected timeline	Actual timeline	Analytical approach	Recruitment outcome
Community asset mapping (CAM) (29)	To document existing assets for maternal-child food security across the West Las Vegas Promise Neighborhood zip codes	The CAM was conducted in four steps: (1) review community documents across five zip codes, (2) engagement of community members in identifying community assets, (3) classify the types of assets providing nurturing care services, and (4) classify assets to nurturing care components (health, responsive caregiving, nutrition, security and safety, and early learning).	No	Community assets providing health and nutrition services for the maternal-child population	The five zip codes in the West Las Vegas Promise Neighborhoods	3 months	5 months (February to June 2022)	Descriptive analysis using the Food Insecurity Index was used to determine food insecurity risk in each zip code. Analyses explored whether disparities exist in nurturing care assets across zip codes with moderate and high food insecurity.	354 community assets providing health and nutrition services for the maternal-child population Findings have been published elsewhere (32).
Maternal-child health survey (MCH)	To document baseline levels of food insecurity, health, and nutrition among pregnant individuals and children under 36 months	The MCH survey consisted of 76 questions for pregnant respondents and 116 for those respondents with children under 36 months. Questions consisted of sociodemographic, validated instruments, and specific maternal-health outcomes. The estimated time to complete was 30–45 min. The survey could be completed in person or virtually in English or Spanish.	Yes: $10 gift card	Pregnant women and caregivers with children under 36 months	500	3 months	5 months (June to October 2022)	Descriptive analyses and the association of food insecurity levels with maternal-child hardship outcomes, such as depression, anxiety, lack of insurance, and exclusive breastfeeding	501 respondents (69 pregnant and 429 caregivers with children under 36 months)
Knowledge, attitudes, practices survey (KAP)	To identify baseline barriers and facilitators on knowledge, attitudes, and practices of perinatal care professionals on food security screening and referrals	The KAP survey consisted of 52 questions, including Knowledge (*n* = 10 items), Attitudes (*n* = 10 items), Practices (*n* = 14 items), and 18 questions related to sociodemographic, experience, and workplace. The estimated time to complete the survey virtually was 15 min.	Yes: $15 gift card	Individuals 18 years of age and older providing direct services to pregnant women and/or children three years or younger at a community organization located in or adjacent to WLVPN, as well as perinatal care professionals (e.g., doulas and mental health practitioners) who travel to WLVPN to provide services.	80–100	2 months	4 months (August to November 2022)	Descriptive analyses of each section KAP were analyzed.	72 practitioners (35 health, 10 nutrition, 7 early learning, 9 responsive care, 11 security and safety) Findings have been published elsewhere (35).
In-depth interviews (IDI)	To investigate the association between perceived discrimination and utilization of perinatal care services targeting pregnant women and caregivers of children under three years old and service practitioners within the West Las Vegas Promise Neighborhood	The IDI questions about perceived discrimination and utilization of perinatal health and nutrition services were tailored to perinatal care practitioners and pregnant women and caregivers. Interviews were conducted virtually in English or Spanish, lasted 20–60 min, were audio-recorded, and transcribed verbatim.	Yes: $15 gift card	Nurturing care practitioners who worked among this population for at least 6 months Pregnant women or caregivers of children up to 36 months	15 perinatal care professionals 10 pregnant women or caregivers	2 months	4 months (November to February 2023)	A phenomenological approach was used to thematic analyze the IDI. The Public Health Critical Race (PHCR) praxis and the Social Ecological Model (SEM) were employed to guide data analyses.	25 interviews (15 nurturing care practitioners and 10 pregnant women or caregivers) Findings have been published elsewhere (33, 34).

Despite these outreach efforts, we encountered several barriers to recruiting participants, which prompted our research team to develop engagement strategies to reach the desired sample size for each data collection method. In this study, we focused on reporting barriers to participant recruitment (phase 1) and practical engagement strategies (phase 2) used for data collection in the maternal-child food insecurity needs assessment. Findings from the maternal-child food insecurity needs assessment have been published elsewhere (32–35).

### 2.3 Reflexivity statement

The EARN-FS research team was led by the principal investigator (PI), who is a new investigator (GB) and a faculty member employed at a minority-serving institution. Additional members included a project manager (CH) who is a well-known maternal-health advocate, a postdoctoral fellow with a background in Anthropology (KH), two faculty members (JF, TG) with experience in CBPR, two faculty physicians working at the academic Pediatrics and Obstetrics clinics (DS, JD), and two representatives of the partner community organization (SH, LG). A senior implementation scientist (AB) and two NIH-project scientists (AB, JC) were also part of the research team, interacting scientifically with the funded cooperative agreement. We acknowledge that our individual characteristics and academic/community affiliations may have shaped the data collection challenges described in this manuscript.

### 2.4 Data sources

In this study, the data sources analyzed were the documentation of barriers, engagement strategies, and outcomes. The EARN-FS research team held weekly meetings throughout the 12 months of data collection. In these meetings, the research team checked progress in achieving the sample size for each data collection method, debriefed about the process by discussing barriers, and defined engagement strategies to be applied in the following week. The practical engagement strategies were defined based on multiple sources, such as the research team’s extensive experience in working with underserved communities and conducting participatory research; a literature review on strategies for engaging communities, overcoming data collection barriers, and managing online surveys; and suggestions from the community partners working with maternal-child populations in Clark County, Nevada.

After each meeting, the project manager (CH) was responsible for documenting in a spreadsheet detailed information on the barriers to recruiting participants, practical engagement strategies, and outcomes for each engagement strategy (i.e., whether it promoted engagement or not). The PI (GB) and the project manager (CH) met weekly to review the spreadsheet, expand notes, and check for consistency. In addition, the spreadsheet was reviewed monthly during the larger EARN-FS team meetings that included community partners, consultants, and funders. This process was repeated throughout the data collection period, which resulted in a comprehensive documentation of barriers and practical engagement strategies.

### 2.5 Data analysis

A two-phase qualitative thematic analysis of the barriers to recruiting participants and practical engagement strategies was conducted at the end of the data collection period. The thematic coding process entailed inductive (i.e., themes to emerge from the data) and deductive (i.e., pre-defined themes based on existing framework) approaches that included individual reviewers coding line-by-line, followed by a comparison of coding among researchers. Any disagreement went through a reflexive analysis and peer debriefing to ensure methodological rigor throughout the process (36, 37).

#### 2.5.1 Phase 1. Barriers to participant recruitment

The goal of this step was to document and systematize the barriers to participant recruitment encountered during data collection. In the first phase of the thematic analysis, data on barriers to recruiting participants were coded line-by-line inductively by two reviewers (CH and GB), which resulted in high-level themes of barriers. These high-level themes were coded deductively by three reviewers (CH, KH, GB) to classify them across the three levels of the socio-ecological model (32). Community-level barriers were defined by the relationships with organizations we worked with in the WLVPN, and included community resources, public policies, and systemic beliefs. The service level was determined by the perspectives, experiences, and beliefs held by practitioners of nurturing care services in the greater WLVPN area. Lastly, the family level was defined by the characteristics, beliefs, cultural norms, and power-related stressors of a family or individual in the WLVPN.

#### 2.5.2 Phase 2. Practical engagement strategies

The goal of this step was to examine the characteristics and effectiveness of the practical engagement strategies used. Thus, in the second phase of the thematic analysis, a similar coding approach was conducted. First, data on engagement strategies were coded line-by-line inductively by two reviewers (CH and GB), which resulted in high-level themes of strategies to overcome the recruitment barriers. Next, three reviewers (GB, CH, KH) used an interpretative approach to name the strategies (e.g., “meeting,” “planned compensation,” etc.), followed by a description of (a) the research team approach/activities, (b) the level of effort required by the research team to implement the strategy (high, medium, low effort), and (c) respective outcomes. Then, (d) the effectiveness of the strategy was assessed based on the degree to which it succeeded in reaching the target population or meeting engagement objectives.

#### 2.5.3 Phase 3. Meaningful community engagement principles

The goal of this step was to examine whether strategies aligned with established principles of meaningful community engagement. In the third phase, the high-level themes of strategies documented in the earlier step were coded deductively by three reviewers (CH, KH, GB) to map them onto the Meaningful Community Engagement Framework by Turin et al. (28). In this study, the framework was slightly adapted by combining “no ‘parachutes in and out’” and “not missing the mass population” principles into a single principle named “building trust and long-term relationships” (28). An operational definition for each of the five principles are provided in Table 2. Thus, during the thematic coding, our analysis mapped the practical strategies within five principles from the Meaningful Community Engagement Framework.

**Table 2:** Adapted definitions of community engagement principles for building partnerships in the EARN-FS study.

Community engagement principles	Adapted definition to the EARN-FS study	Examples of strategies from Turin et al. (28)
Building trust and long-term relationships*	Trust building requires no “parachutes in and out” research, along with consistent, clear, and collaborative communication that facilitates relationships at the family, service, and practitioner levels to create a common understanding of the community ecosystem.	Continue following up with communities to maintain strong relationships Selecting community members broadly (e.g., champions, members with lived experience)
Listening with a blank slate	All conversations must seek to better understand the perceptions and experiences of community members in defining issues of importance to them, not the research team.	Start conversations by asking community members what they think the issues are (instead of assuming the issues)
Planning to compensate for contributions	A community member’s knowledge is expertise and deserves compensation through financial or in-kind incentives.	Through financial or in-kind compensation or benefits
Community service with no strings attached	Research teams need to conduct community service activities that are unrelated to data collection and provide benefits to the community ecosystem with “no strings attached.”	Conduct activities that are beneficial to the community, not only focusing on data collection (e.g., educational workshops, outreach events)
Focus on capacity building	Researchers must provide opportunities to develop skills that allow people and/or organizations to progress through their careers.	Opportunities to develop skills that allow them to progress through their careers

*This principle combines (1) no ‘parachute in and parachutes out’, (2) not missing the mass population from the original Turin et al. (28).

## 3 Results

We recruited 598 participants across the different data collection methods for the maternal-child food insecurity needs assessment (see Table 3). Overall, the demographic characteristics of the participants recruited are similar to the characteristics of the WLVPN. The only exception was the underrepresentation of Black/African American participants in the maternal-child health survey for pregnant women and caregivers of children under 3 years of age. Barriers to participant recruitment (phase 1) and practical engagement strategies (phase 2) used for data collection in the maternal-child food insecurity needs assessment are described in the next sections.

**Table 3:** Demographics of recruited participants.

	Maternal-child health survey for pregnant women and caregivers (MCH)		Knowledge, attitudes, practices survey for professionals (KAP)		In-depth interviews for professionals (IDI)		In-depth interviews for pregnant women and caregivers (IDI)		Total	
Participants	*n* = 501		*n* = 72		*n* = 15		*n* = 10		*n* = 598	
Race	*n*	%	*n*	%	*n*	%	*n*	%	*n*	%
White	390	78.6	27	37.5	4	26.7	5	50.0	426	71.2
Black or African American	63	12.7	14	19.4	5	33.4	3	30.0	85	14.2
Asian	7	1.4	17	23.6	4	26.7	1	10.0	29	4.8
Other /biracial	43	8.7	9	12.5	2	13.4	1	10.0	55	9.2
Ethnicity										
Hispanic/Latino	144	29	22	30.6	3	20	4	40.0	173	28.9

### 3.1 Phase 1. Barriers to participant recruitment

Eleven barriers to participant recruitment were identified: six at the community level, five at the service level, and five at the family level. The three barriers that co-occurred at both the community and family levels were “distrust of researchers,” “population over-surveyed from previous research,” and “fear of mandatory reporting of family issues or due to immigration status.” Table 4 outlines the barriers to reaching the data collection goals across the community, service, and family levels.

**Table 4:** Barriers to community needs assessment data collection across socio-ecological levels.

Barriers	Data collection project	Socio-ecological levels		
		Community	Service	Family
(A) Distrust of the academic institution	MCH, KAP, IDI, CAM	x		
(B) Poor engagement in the advertisement of data collection materials	MCH, KAP, IDI	x		
(C) Short data collection period	MCH, KAP, IDI	x		
(D) Time constraints or disinterest in research participation	CAM, KAP, IDI		x	
(E) Use of non-specific and nondescript terms in advertising	KAP		x	
(F) Getting a hold of organizations and practitioners due to time constraints	CAM		x	
(G) Low participation and response rate	MCH, KAP, IDI		x	x
(H) Distrust of researchers	MCH, IDI	x	x	x
(I) Population over-surveyed from previous research	MCH, IDI	x		x
(J) Low incentive amount due to the time required to complete the surveys	MCH, IDI			x
(K) Fear of mandatory report of family issues or due to immigration status	MCH, IDI	x		x

MCH (Maternal-child health survey), KAP (Knowledge, attitudes, practice survey), IDI (In-depth interviews), CAM (Community asset mapping).

Barriers at the community level included (A) distrust in the academic institution disseminating the surveys to organizations in the WLVPN. During the EARN-FS team interaction with organizations, many reported a history of being over-surveyed in the WLVPN by researchers, organizations, and universities. This history of over-engagement and research without goals or follow-through generated (B) poor engagement in disseminating the surveys. For example, many community members and organizations discussed feelings of apathy toward the surveys and did not want to disseminate them at events or with their organization members. These overarching barriers were exacerbated due to the (C) short data collection period (*n* = 3 months) established for each data collection method during the proposal development. The projected timeline for each data collection method accounted for preexisting partnerships with Nevada Partners Inc. but did not consider the time needed to build partnerships with new organizations focusing specifically on the maternal-child population.

Barriers at the service level included (D) time constraints or disinterest in participating in the Knowledge, Attitudes, and Practice survey (KAP). Particularly, overburdened clinical staff and medical assistants due to staff shortages and high patient loads at the offices throughout the target zip codes could not help disseminate surveys during their current workflows, or would not respond to the KAP. Another barrier was (E) the use of non-specific and non-descript terms in advertising the KAP to physicians. A community representative of a medical chapter in the state warned our research team that the wording “providers” used in the KAP flyers would prevent physicians in the zip codes from responding to the survey, as they do not self-identify as “providers.” To complete the community assets mapping (CAM) the EARN-FS team proposed to contact the 354 services, practitioners, and organizations listed as assets [for details on CAM (29) methods, see Table 1]. One barrier was to (F) get a hold of organizations and practitioners to gather service and referral details such as operating hours, their target population, and the type of services offered. Many organizations were too busy to answer our phone calls and did not have time to call back. In addition, many organizations were no longer in service and some of the services were unlisted and only discovered via “word of mouth.”

Barriers at the family level included (G) low participation and response rate to achieve the goal of 500 maternal-child health surveys completed within three months. Additionally, barriers related to (H) distrust of researchers were amplified by missing information in advertising about the maternal-child health survey, which compromised the legitimacy of the study in the perceptions of community members. For example, the original flyer design failed to include information (e.g., funding organization), leading some community members to interpret it as a scam. Similarly, several community organizations and participants reported not engaging with the maternal-child health survey due to being (I) over-surveyed by previous researchers without receiving any feedback or results after engaging in their research. An additional barrier was related to (J) the low incentive amount provided for completing the maternal-child health survey. Specifically, there were complaints about the disparities in the amount being provided based on the time commitment. For example, in the maternal-child health survey, participants would be paid $10 for completing a 45-min survey, while practitioners were paid $15 to complete a 15-min survey. Finally, (K) the fear of being reported to Child Protective Services due to the responses provided in the maternal-child health survey or due to their immigration status was a concern. This prevented community members from engaging in the project, as some believed that these interactions could lead to family separation or a loss of governmental benefits. Due to these several challenges, we added a statement that no data were shared with any government organizations and opened the maternal-child health survey for as long as data collection would take to reach the sample size, which was five months.

### 3.2 Phase 2. Practical engagement strategies

Fifteen practical engagement strategies were used to overcome barriers to recruiting participants. Table 5 outlines the approach used to implement each practical engagement strategy, research team effort, the outcomes achieved, and whether the strategies were effective in achieving the target population or engagement goals.

**Table 5:** Practical strategies used by the EARN-FS team across the meaningful community engagement framework.

Principles	EARN-FS team goal	Practical strategies	Approach	Research team’s effort	Outcomes	Effectiveness
1. Building trust and long-term relationships*	To leverage the existing partnership within the West Las Vegas Promise Neighborhood (WLVPN) and assets identified through the community asset mapping to build partnerships for maternal-child food security	Meetings	Conducted initial and follow-up one-on-one meetings with organizations, practitioners, and families for relationship-building and buy-in	High effort from the research team. It took several hours of commitment weekly.	Conducted 1–1 meetings with organizations focusing on the maternal-child population in WLVPN (*n* = 41 organizations)	Target reach was achieved as described in the outcomes.
		Continued Communication	Continued communication and transparency in sharing our research goals	Medium effort from the research team. It required a few hours of commitment monthly or bimonthly.		Target reach was achieved as described in the outcomes.
		Engagement in coalitions	Joined forces with community-serving organizations to learn about their offerings or goals	Medium effort from the research team. It required a few hours of commitment monthly or bimonthly.	Engaged in local and statewide coalitions as well as racial/ethnic workgroups to improve relations and understand goals (*N* = 5 coalitions)	Target reach was achieved as described in the outcomes.
		Workgroup for data collection	Developed weekly meetings to strategize on research outreach	High effort from the research team. It required several hours of commitment weekly.	Weekly meetings led by the EARN-FS team with the UNLV health clinics and Nevada Partners for 8 weeks (*n* = 16 meetings)	Target reach was achieved as described in the outcomes.
2. Listening with a blank slate	To learn about issues and problems that were important to WLVPN community members who were pregnant, mothers, or caregivers of children under 36 months	Listening sessions	Conducted listening sessions in English and Spanish to recruit diverse voices and leaders	High effort from the research team. It required several hours of commitment weekly from different members of the team for coordinating scheduling, disseminating, and organizing the sessions.	Listening sessions conducted with only two English speakers participating in one session (*N* = 5 sessions)	Target reach was NOT achieved as described in the outcomes.
		Visual survey of neighborhoods	Drove through the neighborhood to discover maternal-child assets that could not be located or reached by phone or website	Low effort from the research team. It required a few hours to drive by the neighborhoods.	Conducted drive-throughs at different times of the day (*n* = 3 drives)	Target reach was achieved as described in the outcomes.
		Learn from those inside the academic institution	Learned from the director of the Oral History Research Center at UNLV (Dr. White) about her extensive research and knowledge about the Historic West Side of Las Vegas	Low effort from the research team. It required a few hours to coordinate the schedule for a one-time meeting.	Dr. White connected us with community champions to include in our CAB, a documentary series to review, and a history of the reluctance to participate in research with UNLV.	Target reach was achieved as described in the outcomes.
			Invited undergraduate and graduate students who lived within the targeted zip codes to contact our team	Low effort from the research team. It required a few hours to send out emails to various channels at the university.	Several outreach emails sent through diverse university channels Identified one student living in the zip codes to integrate the EARN-FS team and support communication and outreach messages	Target reach was NOT achieved as described in the outcomes.
3. Planning to compensate for contributions	To make consistent efforts to compensate all participants in the research study for their time, including practitioners, community members of the WLVPN, and the organizations that participate in the process	Planned compensation	Planned budget for compensating community members who accept to join the CAB after the community assessment	Low effort from the research team. Funds for the stipend were planned during proposal development.	Provided each community member was a $60 stipend per CAB meeting organized by the EARN-FS team	Target reach was achieved as described in the outcomes.
		Request additional funding	Increased monetary incentives (see Table 1 for reference) for completing the surveys or interviews	High effort from the research team. It required several hours to prepare a proposal to scientifically justify the request for supplemental funds.	Revised the monetary incentives for the next cycle of data collection through a supplement requesting funding	Target reach was achieved as described in the outcomes.
		Flexibility to revise the budget	Planned budget for compensating community organizations as partners (sub-award or consultants)		Readjusted the original budget to allocate funds for an additional 4 maternal-child-focused community organizations as sub-awards or consultants	
4. Community service with no strings attached	To create educational and informational content with “no strings attached” related to pregnancy, childbirth, breastfeeding, and postpartum to help those who are pregnant, mothers, or caregivers know their reproductive rights	Free educational and training resources	Developed and delivered free educational resources related to pregnancy, childbirth, breastfeeding, and postpartum	High effort from the research team. It required several hours of commitment bimonthly for scheduling, disseminating, and organizing the sessions.	Created a “Know Your Rights” event that was conducted quarterly and disseminated across all five zip codes (*n* = 5 events) with low attendance.	Target reach was NOT achieved as described in the outcomes.
			Provided training to practitioners to educate their clients on their reproductive rights	Medium effort from the research team. It required a few hours of commitment monthly or bimonthly to prepare and delivery trainings.	Provided free training for professionals working at United Health Care, members of the Southern Nevada Maternal and Child Health Coalition, and the Family Nurse Partnership Program through our local Health District (*n* = 3 training) with high attendance.	Target reach was achieved as described in the outcomes.
		Community events	Engaged in community service events with maternal-child-focused organizations	High effort from the research team. It required several hours of commitment weekly to coordinate research personnel and sites for outreach.	Participated in community service events such as Human Milk Drive, Breastfeeding events, Step Up for Kids, and Back to School fairs (*n* = 7 events). Low attendance of potential participants.	Target reach was NOT achieved as described in the outcomes.
		Material branding the EARN-FS	Purchased swag such as bibs, toddler plates with sporks, and raffle items (i.e., car seat, lactation pump)	Low effort from the research team. It required one-time justification to scientifically explain the need to readjust the budget to funders.	Granted authorization to purchase swag items through the NIH funding to be able to promote the project in community events Expanded our direct network with community individuals who were pregnant or had a child under 3 years old	Target reach was achieved as described in the outcomes.
5. Focus on capacity building	To encourage local leadership development among organizers and practitioners working with us for data collection	Community Advisory Board (CAB)	Invited community members and leaders to opportunities to help design and provide feedback on the scientific process through the CAB.	Medium effort from the research team. It a few hours weekly to coordinate invitation, and meetings scheduling.	19 individuals and organizations joined the CAB.	Target reach was achieved as described in the outcomes.
			Offered opportunities to present and engage with high-level stakeholders	High effort from the research team. It required a few hours of commitment monthly or bimonthly to prepare and delivery materials, and research personnel time.	Amplified the network and fostered professional development beyond the scope of the project	Target reach was achieved as described in the outcomes.
		Share preliminary findings and results	Provided opportunities to present findings and data collection		The EARN-FS team was given the opportunity to conduct several presentations to coalitions (*n* = 9 presentations) with high attendance	Target reach was achieved as described in the outcomes.

*This principle combines (1) no ‘parachute in and parachutes out’, (2) not missing the mass population from the original Turin et al. (28).

MCH (Maternal-Child Health), KAP (Knowledge, attitudes, practice), IDI (In-depth interviews), CAM (Community asset mapping).

Of the total practical engagement strategies used, nine strategies required high effort from the research team to implement, which generally meant more than one team member dedicating several hours to complete the activity over a certain period. Three strategies that required high effort from the research team and were not effective in engaging the target population or achieving the engagement goal, including “Listening sessions,” “Free educational and training resources,” “Community events” (Table 5).

### 3.3 Phase 3. Meaningful community engagement principles

This section details the fifteen practical engagement strategies across the five principles of the Meaningful Community Engagement Framework: “Building trust and long-term relationships” (*n* = 4 strategies), “Listening with a blank slate” (*n* = 3 strategies), “Planning to compensate for contributions” (*n* = 3 strategies), “Community service with no strings attached” (*n* = 3 strategies), “Focus on capacity building” (*n* = 2 strategies) (Table 5).

#### 3.3.1 Principle 1. Building trust and long-term relationships

Four practical engagement strategies were utilized: (a) meetings, (b) continued communication, (c) engagement in coalitions, and (d) workgroups to strategize about data collection. By using these strategies, the EARN-FS team met with 41 organizations to discuss their work, introduce themselves, disseminate the surveys, and complete on-site visits. We joined five local and statewide coalitions as well as racial/ethnic workgroups to improve relations and understand community goals. Our team held 16 workgroup meetings to strategize on practical engagement, which helped us to achieve the sample goal for the maternal-child health survey.

#### 3.3.2 Principle 2. Listening with a blank slate

Three practical engagement strategies were employed: (a) listening sessions, (b) visual survey of neighborhoods (i.e., systematic observations made from a moving vehicle (38)), and (c) learning from those inside the academic institution who have worked long-term with these communities or those living within the target zip codes. Through these strategies, we held five listening sessions; however, only two women attended. By driving around the targeted zip codes, we were able to discover several maternal-child resources to include in the CAM, which expanded our knowledge about the existing assets in the community. We met with knowledge individuals inside the academic institution who provided us with community champions and documents to review a history of the reluctance to participate in research. Next, we hosted meetings through the diversity and inclusion and first-generation offices at our academic institution, and we recruited and hired a research assistant who lived within the target zip code to support our outreach and communication efforts based on her lived experience.

#### 3.3.3 Principle 3. Planning to compensate for contributions

Three practical engagement strategies employed were (a) planning to compensate all research participants with a gift card of various amounts (refer to Table 1), (b) flexibility to apply for additional funding to supplement the need raised by participants, and (c) flexibility to revise the budget to sustain the CBPR principles. As a result, the EARN-FS team was able to request additional funds through a supplement application to increase research participant incentives from $10 to $25 for a 15-min survey. In addition, we were able to reallocate funds from the original budget to sub-award or pay consultancy fees to four additional maternal-child-focused community organizations supporting the data collection.

#### 3.3.4 Principle 4. Community service with no strings attached

Three practical engagement strategies were employed: (a) the creation of free educational and training resources, (b) attending community events occurring in the WLVPN, and (c) the creation of materials branding with the EARN-FS logo to incentivize participation and engagement during tabling. As a result of these strategies, we hosted five free educational events and three training sessions with practitioners across the target zip codes. We participated in seven community events throughout the data collection period. The face-to-face environment and the materials branding with the EARN-FS allowed us to receive a few additional responses to the surveys and build connections with community members. Participation with one organization created a snowball effect of connection, allowing greater access to the network of nurturing care services and the ability to hand out flyers with the survey link on it. In the end, we expanded our direct network with community individuals who were pregnant or had a child under 3 years old.

#### 3.3.5 Principle 5. Focus on capacity building

Two practical engagement strategies were utilized within this principle: (a) the creation of a community advisory board (CAB) and (b) the sharing of preliminary findings. We invited 32 individuals and organizations to join the CAB, and 19 joined. The first CAB meeting was held in December 2022, and the quarterly meetings included presentations that helped foster professional development beyond the scope of the project. Additionally, we shared preliminary findings by delivering nine presentations throughout the data collection. While these presentations helped to build capacity and knowledge among the maternal-child coalitions, they also illustrated the power of data and were critical to gathering support for the current data collection.

## 4 Discussion

Our study explored the barriers to recruiting participants for a maternal-child food insecurity needs assessment in underserved communities. Using the EARN-FS project as a case study, we identified barriers at the community, service, and individual levels, which were consistent with existing literature on recruiting “hard-to-reach” populations (3, 39). Our work addressed a critical gap by offering practical examples of engagement strategies that effectively mitigated recruitment barriers in maternal-child health research. Although some of the barriers and solutions described may have been context-specific, documenting these engagement strategies through the lens of the Meaningful Community Engagement Framework provided a practical roadmap rooted in equity-oriented research principles. These insights could inform future community-based research projects aimed at reducing maternal-child health disparities.

A primary data collection barrier was the lack of trust due to previous interactions of community members with academics, leading to delays in recruiting participants. To rebuild trust, our yearlong engagement focused on restoring and nurturing relationships by listening with a blank slate—approaching conversations free from prior knowledge, existing interpretations, or judgments. This required cultural humility and adjusting preplanned strategies. Our team dedicated extensive hours to calls, site visits, and coalition meetings, which were not accounted for in the original timeline and required high efforts from the research team. Despite these challenges, these practical engagement strategies were effective in forming partnerships with maternal-child health organizations. Each interaction allowed us to listen, share preliminary findings, and build a shared understanding of maternal-child health needs, fostering community ownership. Therefore, listening with a blank slate was a strategy that supported advancing health equity in our maternal-child health research.

Existing maternal-child health resources in underserved communities, such as the WLVPN, might have been scarce, making engagement more challenging. In this context, the CAM became a key data collection method, identifying both existing and missing maternal-child health resources (32). This process informed strategic partnerships and advocacy efforts. For example, through the CAM, we discovered that several maternal-child health services lacked a physical office or structure in the community; instead, they relied on travel to reach clients within these communities (32). These findings informed our practical engagement strategies, including bringing together community organizations located in the communities and traveling maternal-child health organizations to enhance the availability of maternal-child services in underserved areas. Another practical engagement strategy aimed to build capacity and provide community services with no strings attached. This included providing free educational, training resources, and co-writing grants and applications with community members. As a result, funds to establish several perinatal community services, such as nutrition security community workshops, weekly breastfeeding support groups, and training the lactation workforce and physicians in breastfeeding, filled service gaps and supported community capacity building. Whereas the capacity building strategies required high efforts from the research team, they were effective in achieving engagement goals such as strengthening partnerships and trust among the research team and community organizations.

Another key strategy was ensuring fair compensation for community contributions. Despite the anticipation of compensating those who join the CAB, flexibility in revising the budget allocation and the availability of supplemental funds were practical engagement strategies crucial to enable a fair compensation for contributions. In order to achieve this engagement goal, the strategies used required high effort from the research team with several hours to prepare a proposal to scientifically justify the reallocation of funds and request for supplement funds. Due to these efforts, after listening to the community, our team was able to increase the dollar amount of incentives. Increasing the monetary value of incentives was important and is described as an ethically acceptable tool for promoting recruitment and retention (40, 41). Although the strategies used were effective, our team has since reflected on alternative approaches to ensure fair compensation for community contributions. These include supplementing monetary incentives with other types of rewards, such as purpose-driven (e.g., sense of group mission), social (e.g., making a change to the community), and status-based (e.g., recognition as a community leader) motivations throughout the engagement process (42).

Our findings align with the principles outlined in the Meaningful Engagement Framework, which served as a dynamic roadmap for developing engagement strategies, as suggested by prior study (28). However, some practical strategies required high effort from the research team but were ultimately ineffective in engaging the target population or achieving the intended goals. For example, we attempted to engage community members and university students residing in the targeted zip codes through listening sessions to understand barriers to participant recruitment and invite students with lived experience to be part of the research team, respectively. However, these listening sessions were unsuccessful, with little to no attendance. At the time, our CAB had not yet been established, and it is possible that implementing these strategies later in the project, with advisory board input, might have yielded different outcomes. Conversely, other strategies, such as participating in community events unrelated to maternal and child health and hosting free educational workshops did not directly result in participant recruitment. Nevertheless, they proved valuable in building trust and relationships with community members. These efforts also served as a way to demonstrate the research team’s genuine commitment to the community and its causes.

### 4.1 Limitations

Implementing practical engagement strategies was not without challenges. First, our research team had unique characteristics that may have affected or exacerbated the data collection barriers encountered and must be considered when interpreting the findings. One key factor is that an early-career researcher led the research team. Thus, the overwhelming and time-consuming task of engaging with many new individuals, practitioners, and organizations was particularly daunting to navigate. Additionally, the original research team structure relied heavily on faculty members with expertise in different research methods, and little funding was allocated for community engagement and outreach. However, faculty experts did not have the time to prioritize efforts in community engagement for maternal-child health due to competing academic priorities. This corroborates evidence from prior studies that report challenges in engaging faculty in research at minority-serving institutions due to heavy teaching loads, unrealistic expectations about grants and funding, and developing partnerships to augment research opportunities at non-research institutions (43, 44). Another factor is that the research team is based in a minority-serving institution. In our study, we encountered challenges related to the limited research infrastructure at the funding and academic levels, such as recruiting an interdisciplinary research team to support engagement strategies aimed at building and maintaining trust and relationships (7, 45).

### 4.2 Implications for future research

The practical engagement strategies examined in this manuscript were used as initial engagement efforts during the community needs assessment. A key lesson to improve the quality of future research for vulnerable groups, such as pregnant individuals and children living in underserved communities, is the necessity of allocating time to build research capacity and train teams for effective engagement (8, 39). The amount of time commitment necessary to implement these strategies can be especially challenging in low-resource institutions and for new investigators who are establishing research teams (43). Additionally, various strategies discussed in our research meetings were deemed unfeasible to implement due to a lack of resources, insufficient time, or being inappropriate for achieving the engagement goals. Because of this, our team suggests that future researchers consider categorizing engagement strategies based on their specific goals, such as initial engagement versus continued engagement. For example, in our study, initial engagement efforts enhanced the CBPR approach and facilitated the successful completion of the needs assessment in communities where maternal-child health research had never been prioritized. By the end of the needs assessment timeline, our research team was able to convene a CAB consisting of 19 individuals and organizations meeting quarterly since December 2022 to co-create and implement a multilevel intervention to mitigate maternal-child food insecurity (16). The collaborative process between the research team and the community members during the needs assessment fostered a sense of ownership over the findings from the needs assessment and subsequent intervention development (16), increasing buy-in and participation. To sustain community engagement, our team planned continued/ongoing engagement strategies, including quarterly meetings and a monthly newsletter. These efforts aimed to enhance transparency, strengthen networks, and improve collaboration among the CAB members, which has been described as increasing the likelihood of long-term success in reducing food insecurity (46). Lastly, the lessons learned from developing and implementing practical engagement strategies reflect broader equity research principles applicable to public health practitioners and researchers developing programs to reduce maternal-child health inequities in underserved U. S. communities.

## Data Availability

The original contributions presented in the study are included in the article, further inquiries can be directed to the corresponding author.
